# Ultrasound Indoor Positioning System Based on a Low-Power Wireless Sensor Network Providing Sub-Centimeter Accuracy

**DOI:** 10.3390/s130303501

**Published:** 2013-03-13

**Authors:** Carlos Medina, José Carlos Segura, Ángel De la Torre

**Affiliations:** CITIC-UGR, TSTC. University of Granada, ETSIIT C/Periodista Daniel Saucedo Aranda s/n 18071, Granada, Spain; E-Mails: segura@ugr.es (J.C.S.); atv@ugr.es (A.T.)

**Keywords:** indoor positioning, ultrasound time-of-flight, wireless sensor network, low power

## Abstract

This paper describes the TELIAMADE system, a new indoor positioning system based on time-of-flight (TOF) of ultrasonic signal to estimate the distance between a receiver node and a transmitter node. TELIAMADE system consists of a set of wireless nodes equipped with a radio module for communication and a module for the transmission and reception of ultrasound. The access to the ultrasonic channel is managed by applying a synchronization algorithm based on a time-division multiplexing (TDMA) scheme. The ultrasonic signal is transmitted using a carrier frequency of 40 kHz and the TOF measurement is estimated by applying a quadrature detector to the signal obtained at the A/D converter output. Low sampling frequencies of 17.78 kHz or even 12.31 kHz are possible using quadrature sampling in order to optimize memory requirements and to reduce the computational cost in signal processing. The distance is calculated from the TOF taking into account the speed of sound. An excellent accuracy in the estimation of the TOF is achieved using parabolic interpolation to detect of maximum of the signal envelope at the matched filter output. The signal phase information is also used for enhancing the TOF measurement accuracy. Experimental results show a root mean square error (rmse) less than 2 mm and a standard deviation less than 0.3 mm for pseudorange measurements in the range of distances between 2 and 6 m. The system location accuracy is also evaluated by applying multilateration. A sub-centimeter location accuracy is achieved with an average rmse of 9.6 mm.

## 1. Introduction

In recent years the use of location information and its potentiality in the development of ambient intelligence applications has led to the design of many local positioning systems (LPS) based on different technologies. Systems based on radio frequency signals (RF) require fewer infrastructures than other technologies but have less accuracy. This accuracy is of tens of centimeters for UWB (Ultra Wide-Band) systems based on measurements of TOA (Time-of-Arrival) [1], of several meters using WiFi [2], ZigBee [3] and RFID (Radio Frequency IDentification) [4] or tens of meters for mobile networks [5]. Such precision is unacceptable for applications with centimeter accuracy requirements. Advances in artificial vision can achieve accuracies of several centimeters at the expense of having to use an expensive infrastructure with a low modularity and high processing demand [6].

Unlike these technologies, the ultrasound signal has several advantages such as a slow propagation speed, a negligible penetration in walls and a low cost of the transducers. The characteristics of the ultrasound signal are interesting for use in indoor positioning systems (IPS). The accuracy achieved by ultrasound is typically of a few centimeters. The time-of-flight (TOF) of the signal in its propagation from a transmitter device to a receiver device is used to calculate the distance between them taking into account the propagation speed of sound. This requires a correct temporal synchronization of the network nodes.

The synchronization can be easily achieved through electrical pulses in systems with a wired connection between nodes. ATLINTIDA [7] is an example of this type of systems where the transmitter nodes (with fixed position) are wired to an interface through which they also receive power supply. The receipt of an electrical pulse in the transmitters indicates the beginning of signal transmission. On the other hand, the mobile node is battery operated. This node uses a radio module for transferring the received signal to an AD/DA card connected to a PC that calculates its position. The wired connection complicates the system installation and introduces additional costs in its deployment. This problem is solved using a wireless connection between nodes, although it entails others synchronization problems and requires to change the batteries regularly.

The synchronization problem in wireless sensor networks (WSNs) is usually solved using radio frequency signals (RF). In the literature several examples of positioning systems based on ultrasound and RF can be found. The negligible propagation delay of the RF signal allows one to use this signal for beginning synchronously the transmission and reception processes of ultrasonic signal. Thus, achieving accurate distance estimations (inferred from the TOF of ultrasonic signal) is possible.

For example, Active Bat [8] uses an infrastructure of fixed nodes (beacons) located on the ceiling that operate as ultrasound receivers. The target node (mobile) works as ultrasound emitter and its location is calculated from information of time-of-flight sent by the beacons to a central node. In this system a 433 MHz radio link for providing synchronization information to the network nodes is used. On the contrary, in Cricket system [9] the mobile node works as ultrasonic receiver and the beacons work as ultrasonic transmitters. The ultrasonic pulse transmission is accompanied by an RF pulse to provide the necessary synchronization. The TOF measurements performed at the receiver are returned to a central node through an RF data link where the mobile position is calculated. Unlike the previous systems, Dolphin system [10] uses ultrasonic transducers of greater beam width and bandwidth. Each node has two ultrasonic transducers, one for transmitting and another for receiving, so the system can be configured in two ways: (a) centralized, where the mobile node works as a transmitter and the others nodes as receivers and a central node calculates the mobile position based on TOF measurements returned by the receiver nodes; (b) privacy-oriented, where the mobile node works as a receiver and calculates its own position. The above described systems have a location accuracy of several centimeters.

Newer systems such as 3D-LOCUS [11,12] achieve better accuracy than the above mentioned ones. The evaluation of the 3D-LOCUS system shows sub-centimeter accuracy and a resolution of several millimeters in a controlled environment and a reduced location space. Despite its excellent accuracy, the scalability of this system is limited by the wired connection between the beacons and the central node, with a high deployment cost. Moreover, 3D-LOCUS uses a data interface to provide control information and a different one to provide synchronization, which increases the hardware cost. Its short distance range requires a large number of nodes to cover large location areas. Like most systems described in the literature, 3D-LOCUS offers poor flexibility in modifying signal characteristics in the transmission and reception processes, so it cannot be easily adapted to different applications.

In this paper we present a new IPS called TELIAMADE, originally aimed for use in ambient intelligence applications for the care of dependent people with reduced mobility. However, this system could be used also in industrial environments to accurately control the movement of machinery. With this system, we aim to provide a new low-power wireless architecture, greater accuracy in TOF measurement and high configuration-flexibility.

TELIAMADE has a master-slave topology with a coordinator node and a set of end nodes. The communication between nodes is based on the exchange of radio messages using the ZigBee protocol. The network administrator can configure the nodes operation mode by sending ZigBee messages through the coordinator node, which acts as a gateway between the user application and the end nodes. The availability of a module for transmitting and receiving ultrasonic signal in each end node enables them to operate as ultrasonic transmitters or receivers. Some transmission and reception parameters such as the size and signal duration, the sampling frequency or the measurement rate can be configured. This way, the system operation can be adjusted to scenarios with different requirements.

The TELIAMADE system makes use of a shared radio link for data transmission and network synchronization purposes. The negligible propagation delay of the radio messages enables to use the ZigBee interface to provide synchronization information to the network nodes. TOF measurements are performed periodically following a TDMA scheme, which enables an access to the ultrasonic channel without the need of a supervisor node to monitor the process. However this measure scheme entails some synchronization problems that must be avoided in order to correctly measure the time-of-flight of ultrasound signal.

The TOF measurement is estimated using a quadrature detector in which quadrature band-pass sampling is used in order to optimize memory requirements and reduce the computation cost in signal processing. In order to get an accurate TOF estimation, we use parabolic interpolation to precisely detect the envelope maximum at the quadrature detector output. The signal phase information is also used to improve the TOF estimation, thereby increasing the system accuracy by using multilateration from the distances derived from the TOF measurements.

The paper is organized as follows. In Section 2 the TELIAMADE system architecture is described. In this section the hardware design of the nodes is shown as well as the way in which the ultrasound signal is generated and detected. Section 3 describes the algorithm to measure the TOF of ultrasonic signals. In Section 4 we describe the distance estimation from the TOF measurement and the positioning algorithm. In Section 5 results of the system accuracy of the distance measure are shown. Results on location accuracy of the system are provided making use of multilateration. Finally, in Section 6 we summarize the main conclusions of this work.

## 2. System Description

**Figure 1.**
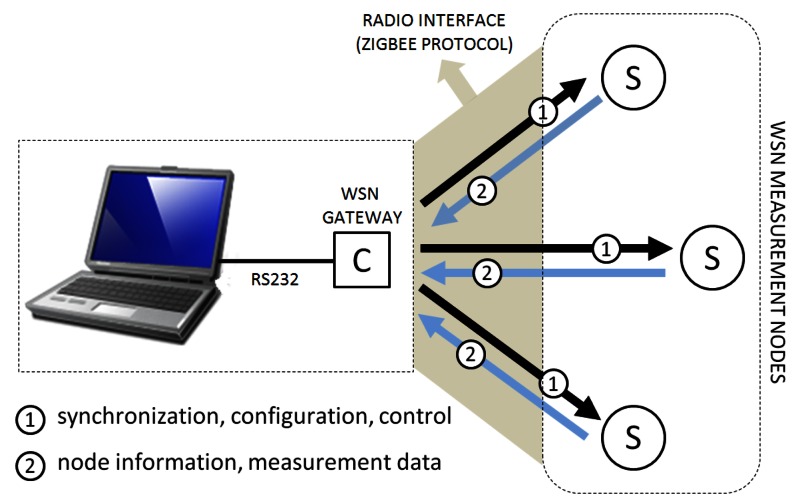
Illustration of the connection between nodes in the TELIAMADE system based on a master-slave topology. A coordinator node connected to a PC is operating as master and allows synchronization, configuration and control of the network end nodes. The end nodes operate as slaves. These can send information about their configuration and also data about TOF measurements of the ultrasound signal when they are operating as ultrasonic receivers, which are processed on a PC. The communication between nodes is based on the exchange of radio packets using the ZigBee Protocol.

TELIAMADE has a star network topology in which a master node (coordinator) manages a set of end nodes using a digital radio link based on the ZigBee protocol [13]. The end nodes are equipped with a low-power microcontroller (PIC18F4620) [14] and a radio chip (CC2420) [15] that implements the physical layer of the IEEE 802.15.4 standard. The other layers of the ZigBee protocol stack are implemented by software in the microcontroller. Figure 1 shows the connection of the different elements that make up the TELIAMADE system. The coordinator node is physically connected to a PC through a serial interface. This node works as a gateway between the user application and the network nodes. Control information can be sent to the nodes for changing their configuration and to monitor their operation using commands.

The nodes are also equipped with a pair of ceramic ultrasonic transducers (400ST/R120) [16] with a resonance frequency of 40 kHz and a 6 dB bandwidth of 2 kHz. Therefore, each node can be configured as an ultrasound transmitter or receiver. The TOF of signal is obtained by measuring the propagation delay of an ultrasound pulse from a transmitter node to receiver one. The TOF estimation is performed at the receiver node.

The generation of ultrasonic signal is performed using the EUSART (Enhanced Universal Synchronous Asynchronous Receiver Transmitter module) of the microcontroller. A sequence of alternating 0 and 1 bits is generated at a baud rate equal to twice the resonance frequency of the transducer (*i.e.*, 80 kbps) with an appropriate duration. For example, a signal burst of 1 ms requires the transmission of an alternating sequence of 80 bits. The EUSART output is buffered in digital inverter gates (MC14049UB) [17] implementing a push-pull configuration in order to provide a higher gain in the signal transmission. A signal amplitude of 2*V_DD_* is achieved when the node is powered with *V_DD_* volts. Since signal generation is digital, it is possible to use a coded signaling with different modulation techniques (e.g., BPSK) for providing greater robustness against noise and multipath effects.

The TOF computation is performed using a quadrature digital detector. The received signal from the ultrasonic transducer is amplified and filtered using an active second order analog filter with a 40 kHz center frequency and a Q = 8 quality factor. Its implementation requires the use of a dual operational amplifier (LMC6482IN) [18] and several passive components (resistors and capacitors). This filtering is very important to reduce the out-of-band noise. The conditioned analog signal is then sampled and stored in a memory buffer. A band-pass sampling scheme [19,20] is used to reduce the required memory and processing resources.

**Figure 2.**
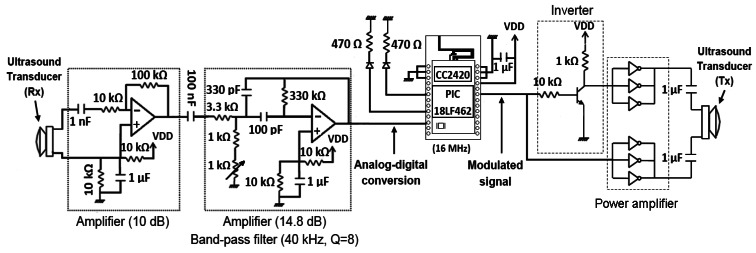
Hardware design of the TELIAMADE nodes. In transmission, the ultrasound signal is amplified before being driven to the transducer, in order to increase the signal power and improve the range of the system. In reception, the signal is amplified and band-pass filtered before being sampled and digitized using the A/D converter of the microcontroller.

**Figure 3.**
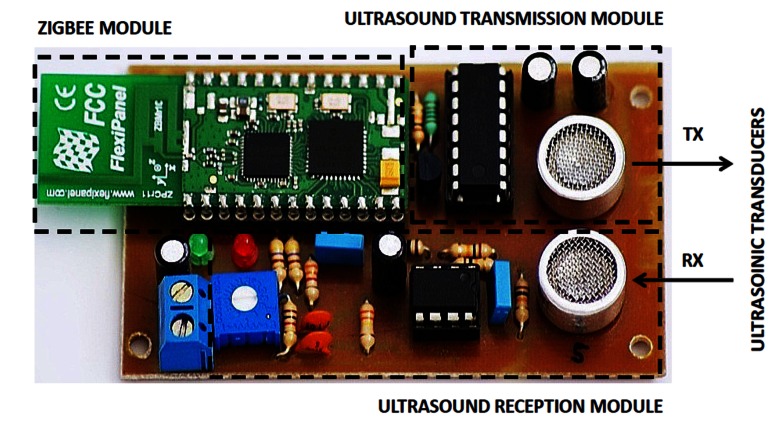
Picture of a TELIAMADE node. The regions identified by dashed line show the hardware components of the radio module and the signal conditioning modules for transmission and reception of ultrasonic signal.

The signal sampling is done using the A/D converter of the microcontroller with 10-bit resolution for a suitable dynamic range. The signal samples are stored in the microcontroller RAM memory using 2 bytes per sample. Figure 2 shows the hardware design of the TELIAMADE nodes. Details of the signal-conditioning modules previously described for the transmission and reception of ultrasonic signal can be seen. Figure 3 shows a picture of a TELIAMADE node.

**Figure 4.**
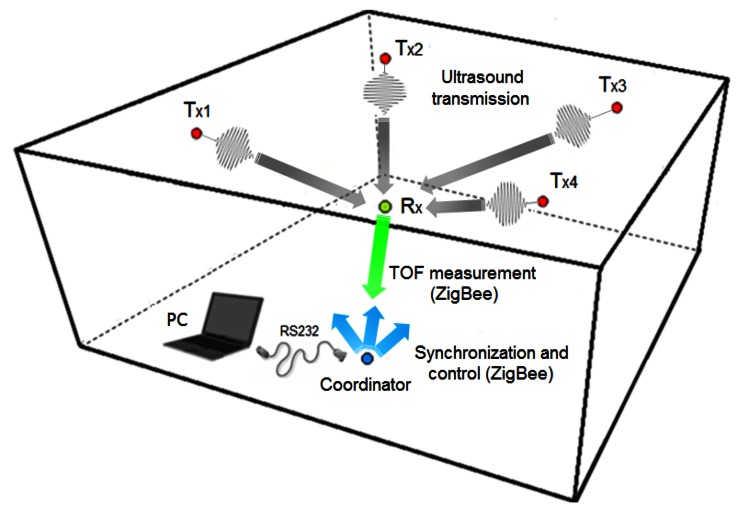
The typical configuration of the TELIAMADE system. A coordinator node is connected to a PC through which all other nodes of the network are managed. A set of fixed nodes work as ultrasonic transmitters (Tx) and are placed on known positions (typically at the ceiling). The mobile node operates as ultrasonic receiver. Its position is determined by applying multilateration using the estimated distances to the transmitter nodes.

The operation of the TELIAMADE system in its typical configuration is shown in Figure 4. The devices located on the ceiling (denoted by Tx) represent the end nodes configured as ultrasonic transmitters. The mobile node (denoted by Rx) is configured as ultrasonic receiver. All nodes in the network are time synchronized by periodic sending ZigBee synchronization messages issued by the coordinator. The TOF measurements of ultrasound signal between transmitters and receiver node are returned to the coordinator to determine the receiver position. Although in the current implementation the receiver position calculation is performed on the PC using multilateration, the calculation capacity of the end nodes allows doing it in the mobile node. This would ensure the privacy of the positioning information as well as the scalability of the system regardless of the number of mobile nodes in the network.

The power consumption of the nodes is mainly due to the radio chip and the microcontroller. The use of the ZigBee protocol enables the radio chip to be configured in a low-power mode. In a network with a large number of distributed transmitter nodes over an area of location, only those nodes closest to the mobile node are useful to estimate its position. In this way, those nodes out of the reception range of the mobile node can be set in a low-consumption mode. In normal mode (A-mode), the radio chip is on and the peripherals of the microcontroller are enabled for processing any service interruption. The radio chip and peripherals are disabled when the node is put in low consumption (B-mode). Included information in synchronization and control packets can modify the operating mode of the radio chip. In this case, the node is periodically awakened to receive the synchronization message and to process this information, using the watchdog timer of the microcontroller. The node is set again to low-consumption mode if the synchronization information indicates that must be set to B-mode. In A-mode, the average consumption of an end node is 29.9 mA and 26.9 mA when operating as ultrasonic transmitter or receiver respectively. This consumption is reduced to 1.7 mA when the node operates in B-mode. This consumption corresponds to a rate of 5 measurements per second. Experimentally it has been shown that a lower measurement rate reduces the consumption although slightly.

The TELIAMADE nodes are powered by AA batteries with a typical capacity of 2,000 mAh, so that the battery life in A-mode would be approximately 67 hours, whereas in B-mode would be 1,176 hours. In a real-life scenario both modes are possible, so that the battery life will depend on the application implemented by the system.

However this consumption can be further reduced. For this we propose a periodic scheduled measurement scheme based on a time division multiple access. Using this approach, the network nodes can be programmed to start transmission or reception of ultrasonic pulses at given time instants. The TDMA scheme enables an orderly access to the ultrasound channel of the transmitter nodes, avoiding collisions in the signal transmission. This synchronization proposal has several drawbacks that need to be addressed to obtain a high accuracy on the TOF estimation. In [21] some of these drawbacks and the strategies adopted to overcome them are described. The nodes can be set in a low-consumption mode during those periods in which a measurement has not been scheduled. In this way the radio module can be awakened just before receiving the synchronization message and returned to low-consumption mode just after. Therefore, the transmitter nodes can maintain its radio module in B-mode during the entire period of measure except for a few milliseconds. In this case, the battery life would be 436 hours (approximately 18 days), assuming that the node operates daily 16 hours in A-mode and 8 hours in B-mode. The consumption of the receiver node is less problematic since changing batteries is easier.

In the next section we describe the procedure used to obtain the TOF measurements of the ultrasonic signal. From this measure, it is possible to estimate the distance between nodes by considering the propagation speed of sound.

## 3. Ultrasonic TOF Estimation

The TOF measurement is obtained from the recorded signal samples in receiver node. The memory capacity (*S*) required to store the signal samples can be estimated as follows. Signal transmission and reception processes start at the same time, therefore the required memory size can be expressed in terms of the maximum signal propagation delay (*τ_max_*) between two nodes, the duration of the transmitted burst (*T_b_*), the number of bytes (*k*) to represent each sample and the sampling frequency (*F_s_*).

(1) $$S=k\cdot F_{s}(\tau_{\max}+T_{b})$$

Low-pass sampling of the ultrasonic signal requires a sampling frequency of *F_s_* ≥ 2(*F*_0_ + *B*), where *B* is the bandwidth and *F*_0_ the carrier frequency. In our case, a sampling frequency of *F_s_* ≥ 84 kHz is required for a signal bandwidth of *B* ∼ 2 kHz and a carrier frequency *F*_0_ = 40 kHz. On the other hand, it is known that signal propagation delay is approximately 3 ms/m for a typical sound speed of 340 m/s. Assuming the maximum distance between nodes is 10 m (*τ_max_* = 30 ms), a burst duration of *T_b_* = 1 ms (transmission of a single pulse) and the use of two bytes to represent each sample (*k* = 2), the memory size required to store the signal samples would be of S = 5,208 bytes. However this approach is not viable because the microcontroller RAM memory is only of 2 kbytes.

A band-pass sampling scheme is implemented to deal with this problem. The general theory of band-pass sampling determines that a band-pass signal with central frequency of *F*_0_ and *W* = *2B* bandwidth (double sideband) can be retrieved from its samples using a sampling frequency of *F_s_* ≥ 2*W*. This sampling frequency is generally lower than the frequency of the carrier (*F_s_* ≤ *F*_0_). The quadrature sampling is a particular case of band-pass sampling. In this case the sampling frequency is conditioned by the bandwidth of the modulating signal [22]. In our system the signal bandwidth does not exceed 2–3 kHz, therefore the use of low sampling frequencies is possible.

With this approach it is possible to handle signals of greater duration to increase the distance range of the system (a smaller sampling rate enables a greater time-of-flight of the signal) or to reduce the processing resources at the receiver to operate with a smaller number of signal samples. Below we will describe the way in which the TOF measurement is estimated by applying a quadrature digital detector using a band-pass sampling scheme.

### 3.1. Quadrature Sampling Detector

The general expression of a bandpass signal *x*(*t*) is given by

(2) $$\begin{array}{l} x(t)=A(t)\cdot \cos(2\pi F_{0}t+\phi (t))\\ =A(t)\cdot \cos(\phi (t))\cos(2\pi F_{0}t)-A(t)\cdot \sin(\phi (t))\sin(2\pi F_{0}t)\\ =I(t)\cdot \cos(2\pi F_{0}t)-Q(t)\cdot \sin(2\pi F_{0}t) \end{array}$$

where the parameters *A*(*t*), *ϕ*(*t*), *I*(*t*), and *Q*(*t*) are referred as signal amplitude, phase, and in-phase and quadrature components, respectively. The signal *x*(*t*) is sampled at the receiver using a sampling frequency (*F_s_*) [22] given by

(3) $$F_{s}=\frac{4F_{0}}{\left(2M-1\right)}$$

where *F*_0_ is the carrier frequency and *M* is any integer. The sampling frequency must meet the condition *F_s_* ≥ *AB*; hence the M parameter can take a value between 3 and 9. The available bandwidth using *M* = 9 is the limit for the bandwidth of the used transducers. The analog to digital converter (A/D) provides digitalized values from *x*(*t*) with a sampling rate of 2*W*(> 2*B*). If we consider a signal *x*(*t*) without energy out of a limit bandwidth *W*, all signal information is contained in a sequence of samples taken at a uniform rate greater than *2W*. The signal *x*(*t*) may be exactly reconstructed using interpolation

(4) $$x(t)=\sum_{n}x(t_{n})\cdot h(t-t_{n})$$

where *t_n_* = *n*/*F_s_* and *F_s_* = 2*W*. The *F*_0_ value can be obtained from Equation (3) using a sampling frequency of *F_s_* = 2*W*. In this way, for values of *F*_0_ = (2*M* − l)*W*/2 the I and Q components can be retrieved from the sampled signal values. Assuming *t* = *t_n_*, the discretized expression of the sampled band-pass signal is as follows

(5) $$x(t_{n})=I(t_{n})\cdot \cos(\pi n(M-\frac{1}{2}))-Q(t_{n})\cdot \sin(\pi n(M-\frac{1}{2}))$$

where *n* identifies a digitized sample. The estimation of the *I* and *Q* components in Equation (5) requires an interpolation process of the signal samples. The signal samples correspond to the interleaving of the in-phase and quadrature components. The sign of these components is conditioned by the selected *M* value. Since the signal cycle is repeated periodically every 4 samples, Equation (5) can be formulated as

(6) $$x(t_{n})=\{\begin{array}{ll} +I(t_{n}) & \text{if}\mathrm{mod}(\text{n},4)=0\\ +Q(t_{n}){\left(-1\right)}^{M} & \text{if}\mathrm{mod}(\text{n},4)=1\\ -I(t_{n}) & \text{if}\mathrm{mod}(\text{n},4)=2\\ -Q(t_{n}){\left(-1\right)}^{M} & \text{if}\mathrm{mod}(\text{n},4)=3 \end{array}$$

where mod(*n*, 4) is the remainder of (*n*/4). Using a value of *M* = 5 and a carrier frequency of *F*_0_ = 40 kHz, the sampling frequency is *F_s_* = 17.78 kHz with an available bandwidth of *B* = 4.44 kHz (Note that *B* < *W*/2 = *F_s_*/4). Similarly, for *M* = 7 the sampling frequency is *F_s_* = 12.31 kHz with an available bandwidth of *B* = 3.08 kHz. For *M* = 9, *F_s_* = 9.41 kHz and *B* = 2.35 kHz.

This invites us to implement a coherent detector based on Equation (5), since alternate samples of signal correspond to the *I* and *Q* components. Because of interlaced sampling, the *I* samples are only known for even values of *n*, while *Q* samples are known for odd values of *n*. However, since both signals are sampled correctly, it is possible to interpolate their values to the nominal frequency *F_s_* using interpolation. In this way the *I* and *Q* components are known at the same instant. The value of the unknown component at *t* instant is calculated by performing an interpolation at *t* = *t_m_* + 1/(4*F*_0_) (displacement of one sample) with *t_m_* = *t_n_* − *N*/2*W*. The signal *x*(*t*) may be exactly recovered from samples according to Equation (4) involving a summation over infinite limits. In practice we are restricted to an algorithm with finite terms. Specifically, the coherent detector involves the summation of Equation (4) containing 2*N* + 1 terms (*i.e.*, *n* = [−*N*, *N*]).

(7) $$\hat{x}(t)=\sum_{n=-N}^{N}x(t_{n})\cdot h(t-t_{n})$$

The *I* and *Q* components are obtained from the signal samples using a pair of quadrature filters derived from an interpolation low-pass filter. This also makes it possible to reduce the out-of-band noise when it occupies a bandwidth *W* ≥ *B*. The selected low-pass filter has an expression similar to the one used in [23], considering a bandwidth *B* = *W*/2. Assuming *t_m_* = 0 and *F*_0_ ≫ *W*, its expression is given by

(8) $$\begin{array}{cc} h(t_{n}) & =\text{sinc}{\left(2B\frac{q}{p}t_{n}\right)}^{p}\cdot \text{sinc}\left(Bt_{n}\right)\\ & =\text{sinc}{\left(n\frac{q}{2p}\right)}^{p}\cdot \text{sinc}\left(\frac{n}{4}\right) \end{array}$$

with *q* = 1 − *B*/*W* and *p* = Int(*Nqπ*/e), where e is the base of the natural logarithm. From Equation (8) we obtain a pair of band-pass quadrature filters multiplying by the cosine and sine functions of frequency *F*_0_ = (2*M* − 1)*W*/2.

(9) $$\begin{array}{l} h_{i}(t_{n})=h(t_{n})\cdot \cos\left(2\pi n\frac{F_{0}}{F_{s}}\right)=h(t_{n})\cdot \cos\left(\pi \frac{n}{2}\right)\\ h_{q}(t_{n})=h(t_{n})\cdot \sin\left(2\pi n\frac{F_{0}}{F_{s}}\right)=h(t_{n})\cdot \sin\left(\pi \frac{n}{2}\right)\cdot {\left(-1\right)}^{M-1} \end{array}$$

These filters provide the values of the in-phase and quadrature components in the instants where they are unknown. Experimentally we have found that 7 coefficients (*N* = 3) are enough to get a good estimation with the minimum computational cost. In order to simplify the calculation of the operations and reduce the memory requirements, the coefficients of the filters are quantized with 3 bits, thus avoiding floating point operations in the microcontroller. The values of these coefficients are:

(10) $$\begin{array}{l} \begin{array}{l} c_{hi}=[\begin{array}{lllllll} 0 & -4 & 0 & 8 & 0 & -4 & 0 \end{array}]\\ c_{hq}=[\begin{array}{lllllll} 1 & 0 & -7 & 0 & 7 & 0 & -1 \end{array}] \end{array} \end{array}$$

**Figure 5.**
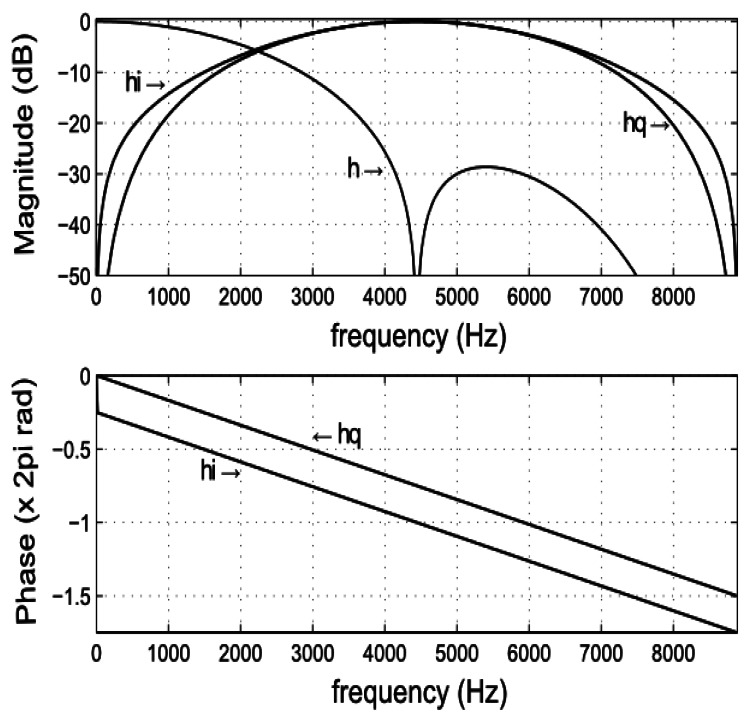
The normalized frequency response of the filters using a value of M = 5 (*F_s_* =17.78 kHz). (Above) Magnitude response of the low-pass prototype filter (*h*) and the band-pass in-phase (*h_i_*) and quadrature (*h_q_*) filters derived from it. (Below) Phase response of the band-pass in-phase and quadrature filters.

Figure 5 shows the frequency response of the quadrature filters using a value of *M* = 5. Despite the quantization of the filter coefficients, we notice that the phase response and the common frequency response are correctly preserved into a bandwidth of 4 kHz around the carrier frequency. Using Equations (7) and (10), the in-phase *x̂_i_*(*t_n_*) and quadrature *x̂_q_*(*t_n_*) filtered signals will be given by

(11) $$\begin{array}{l} {\hat{x}}_{i}(t_{n})=8x(n)-4(x(n-2)+x(n+2))\\ {\hat{x}}_{q}(t_{n})=7(x(n+1)-x(n-1))+x(n-3)-x(n+3) \end{array}$$

Table 1 shows the way in which the *Î*(*t_n_*) and *Q̂*(*t_n_*) components are estimated using Equation (11). In this case we assume an odd value for M in order to avoid a change of sign in the quadrature component. The *m* parameter of the table represents the index of the sample (*n*) expressed in modulus 4.

For transmission of an ultrasound pulse (signal burst of one bit), the in-phase and quadrature components at the output of the matched filter are obtained as

(12) $$\begin{array}{ll} C_{i}(t_{n})=\sum_{k=0}^{K-1}\hat{I}(t_{n}-k) & C_{q}(t_{n})=\sum_{k=0}^{K-1}\hat{Q}(t_{n}-k) \end{array}$$

where *K* = (*T_b_* · *F_s_*) identifies the number of samples of the transmitted signal and *T_b_* is the pulse duration (e.g., 1 ms). The signal envelope is calculated using Equation (12) in the following way

(13) $$C_{e}(t_{n})=\sqrt{C_{i}^{2}(t_{n})+C_{q}^{2}(t_{n})}$$

**Figure 6.**
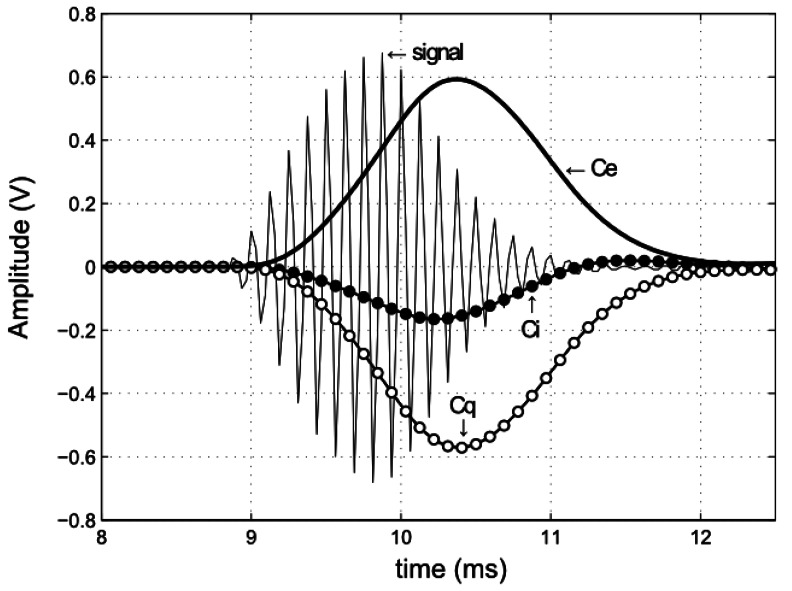
Reception of an ultrasonic pulse emitted by a transmitter node located at a distance of 3 m from the receiver node. The thin continuous line represents the received sampled signal using a sampling frequency of *F_s_* = 32.00 *kHz* (*M* = 3). The curves of filled and empty points correspond respectively to the in-phase (*C_i_*) and quadrature (*C_q_*) components obtained at the output of the matched filter. Finally, the thick continuous line represents the signal envelope (*C*_e_).

Figure 6 shows an example of the curves obtained for the *C_i_*(*t_n_*) and *C_q_*(*t_n_*) components at the output of the matched filter, when an ultrasonic pulse is transmitted. Therein, the signal envelope *C_e_*(*t_n_*) obtained from Equation (13) is also shown. The time-of-flight of the ultrasonic pulse is estimated as the time instant at which the envelope reaches its maximum value. This time is calculated from the index of sample at which the envelope reaches its maximum amplitude (*n_max_*) for a known sampling rate. Therefore the system accuracy is limited by the selected sampling frequency. A maximum error of half a sample can be made. For example, with *F_s_* = 17.78 kHz (for M = 5) the maximum error at the TOF measurement will be 28.12 *μ*s, which is equivalent to a distance error of 9.56 mm assuming a speed of sound of 340 m/s, whereas with *F_s_=* 12.31 kHz (for M = 7) the error will be 13.81 mm. In order to reduce this error we use parabolic interpolation in detecting the envelope peak. It is described below.

### 3.2. Parabolic Interpolation

**Figure 7.**
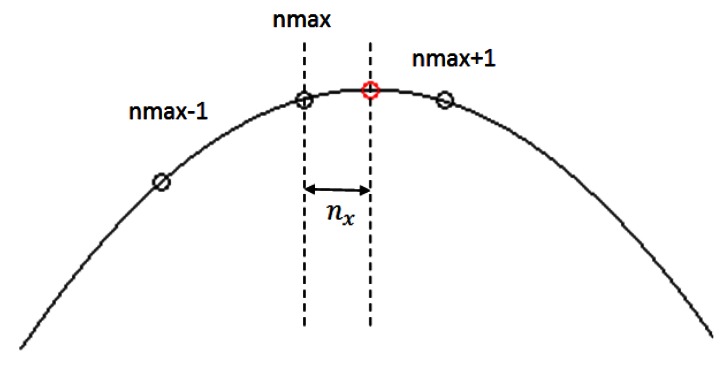
Parabolic interpolation using a buffer size of 3 samples to obtain the maximum of the signal envelope.

The estimation error in determining the envelope maximum is reduced by applying parabolic interpolation considering the adjacent samples around the selected peak [24]. The TOF measurement is calculated as

(14) $$\text{TOF}=\frac{n_{\text{max}}+n_{x}}{F_{s}}$$

where *n_x_* is the fractional correction term obtained from parabolic interpolation. Experimentally we have found that a buffer size of 3 samples is enough to get a good interpolation (see Figure 7). Its value is given by

(15) $$n_{x}=\frac{C_{e}(t_{n_{\text{max}}-1})-C_{e}(t_{n_{\text{max}}+1})}{2\cdot [C_{e}(t_{n_{\text{max}}-1})-2C_{e}(t_{n_{\text{max}}})+C_{e}(t_{n_{\text{max}}+1})]}$$

The interpolation correction leads to errors less than half sample period and reduces the standard deviation of the measurements. Such accuracy enables the use of phase signal information to improve the TOF measurement. Below it is detailed how the phase information can be used to improve the TOF estimation.

### 3.3. Using Phase Information

The signal phase information has a greater resolution and its value is not limited by the used sampling frequency [25]. However it is ambiguous, *i.e.*, the phase is periodic with a period equal to a wavelength. Therefore, the estimated delay from the phase allows us to know the TOF value but it does not specify an integer number of wavelengths. This integer number of wavelengths is estimated using parabolic interpolation as described above. So the phase information provides the fractional correction in the TOF measurement.

This approach is based on the assumption that the signal delay is estimated correctly as a whole number of wavelengths. According to the characteristics of our digital detector, the phase is easily calculated from the in-phase and quadrature components at the output of the matched filter

(16) $$\alpha =\text{atan}2\left(\frac{C_{q}(n_{\text{max}})}{C_{i}(n_{\text{max}})}\right)$$

where atan2 is the four-quadrant inverse tangent. Considering the correction provided by the phase information, the TOF measurement is finally calculated as

(17) $$\text{TOF}=\text{Int}\left[\frac{\left(n_{\text{max}}+n_{x}\right)}{F_{s}}\right]+\frac{\alpha }{2\pi F_{0}}$$

## 4. Estimation of Distances and Positioning

The distance between two nodes is estimated from the TOF measurement using the propagation speed of sound (*c*) as

(18) d=c⋅TOF

To achieve a good accuracy in the distance calculation, it is necessary to properly estimate the speed of sound. The speed of sound in air depends on factors such as temperature, relative humidity, pressure, or air turbulence. In this work we make use of the approach proposed by [26], where only the influence of temperature is considered. The speed of sound (in m/s) is estimated as

(19) $$c\approx 20.06\cdot \sqrt{T+273.15}$$

where T is the temperature in degrees Celsius.

The approach described in this section assumes a proper synchronization of the network nodes, as discussed in Section 1. The TOF measurement requires a precise synchronization between the receiving and transmitting processes of the ultrasound signal. Each network node has a local clock that controls its operation. Clock drifts introduce a delay between the receiver and transmitter nodes and consequently an inaccurate estimation of the TOF value. In a wireless sensor network, such synchronization of the clocks is particularly complex. The details of this network synchronization mechanism can be found in [27].

Assuming a typical location scenario with a mobile node and a set of fixed transmitter nodes, the mobile position can be calculated by applying multilateration using the estimated distances from the receiver node to each transmitter node, which are denoted as *d_i_*, the subscript *i* refers to each transmitter node and takes values within the range *i* = *1*…*N*, where *N* is the total number of transmitter nodes. The transmitter node position is known and it is denoted as (*x_ti_*, *y_ti_*, *z_ti_*). The mobile node position is denoted as *m* = (*x_r_*, *y_r_*, *z_r_*). A system of equations is constructed using the formula of the surface area of a sphere by assuming that the point where the mobile node is located belongs simultaneously to the spheres determined by the fixed nodes (with center in their respective positions and radius equal to the distance to the mobile node). The mobile position can be determined using the least squared method for minimizing the following cost function

(20) $$F={\sum_{i=1}^{N}\left[\sqrt{{\left(x_{ti}-x_{r}\right)}^{2}+{\left(y_{ti}-y_{r}\right)}^{2}+{\left(z_{ti}-z_{r}\right)}^{2}}-d_{i}\right]}^{2}$$

Instead of applying an iterative algorithm to determine the mobile position (*m*), we prefer to use an alternative approach by transforming the non-linear system of equations into a linear system of equations. To do this, a translation of the coordinate system is performed by referencing the position of the first transmitter. This system can be expressed in matrix form as

(21) A⋅m′=b

where:

$$\begin{array}{c} A=\left[\begin{array}{ccc} x_{t1}-x_{t2} & y_{t1}-y_{t2} & z_{t1}-z_{t2}\\ x_{t1}-x_{t3} & y_{t1}-y_{t3} & z_{t1}-z_{t3}\\ x_{t1}-x_{t4} & y_{t1}-y_{t4} & z_{t1}-z_{t4}\\ & \vdots & \\ x_{t1}-x_{tN} & y_{t1}-y_{tN} & z_{t1}-z_{tN} \end{array}\right]\\ b=\left[\begin{array}{c} 0.5\cdot (x_{t1}^{2}-x_{t2}^{2}+y_{t1}^{2}-y_{t2}^{2}+z_{t1}^{2}-z_{t2}^{2}+d_{2}^{2}-d_{1}^{2})\\ 0.5\cdot (x_{t1}^{2}-x_{t3}^{2}+y_{t1}^{2}-y_{t3}^{2}+z_{t1}^{2}-z_{t3}^{2}+d_{3}^{2}-d_{1}^{2})\\ 0.5\cdot (x_{t1}^{2}-x_{t4}^{2}+y_{t1}^{2}-y_{t4}^{2}+z_{t1}^{2}-z_{t4}^{2}+d_{4}^{2}-d_{1}^{2})\\ \vdots \\ 0.5\cdot (x_{t1}^{2}-x_{tN}^{2}+y_{t1}^{2}-y_{tN}^{2}+z_{t1}^{2}-z_{tN}^{2}+d_{N}^{2}-d_{1}^{2}). \end{array}\right] \end{array}$$

The closed solution for *m* by using the least squares method is finally

(22) $$m={\left(A\prime \cdot A\right)}^{-1}\cdot (A\prime \cdot b)$$

## 5. Experimental Results

This section discusses the TELIAMADE system accuracy In Section 5.1, we show some results of the system accuracy in the distance measurement. Although these results are adequate to show the good performance of the system, it is also true that they do not actually reflect the location accuracy in a real environment. Section 5.2 describes the scenario where some position measurements are taken to evaluate the location accuracy of our system in a real environment and the corresponding results.

### 5.1. Accuracy in Distance Measurement

**Figure 8.**
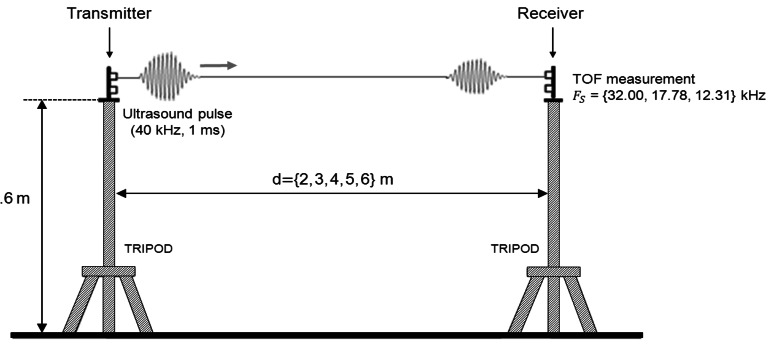
Illustration of the test-bed used in the distance measurements. A transmitter node and a receiver node are placed one in front of the other using a pair of tripods at a height of 1.6 m above the ground. Different distances in the range of 2–6 m are considered. The distance between nodes is set by using a commercial laser rangefinder of precision ±1.5 mm. The transmitter node sends periodically an ultrasound pulse of 1 ms by allowing 8 TOF measurements every 3.2 seconds. The receiver node is configured to use the sampling frequencies of 32 kHz, 17.78 kHz and 12.31 kHz. A total of 500 TOF measurements were taken for each distance and each sampling frequency.

In order to evaluate the accuracy of the system in the distance measurement, we used a pair of nodes (transmitter and receiver) separated by certain known distance. Figure 8 shows the test-bed used in the distance measurements. The transmitter node was configured to transmit an ultrasonic pulse of 1 ms duration. The measurement rate was selected to make a total of 8 TOF measurements every 3.2 seconds. The distance between nodes was inferred from the TOF measurement using the speed of sound from Equation (19). Since the average temperature was 24.3 °C, a speed of sound of 345.52 m/s was estimated. The receiver node was separated from the transmitter node in steps of 1 m in the range from 2 to 6 m. A total of 500 TOF measurements were taken for each distance. A commercial laser rangefinder (BOSCH-DLE70) of precision ±1.5 mm was used to measure the separation between nodes.

**Figure 9.**
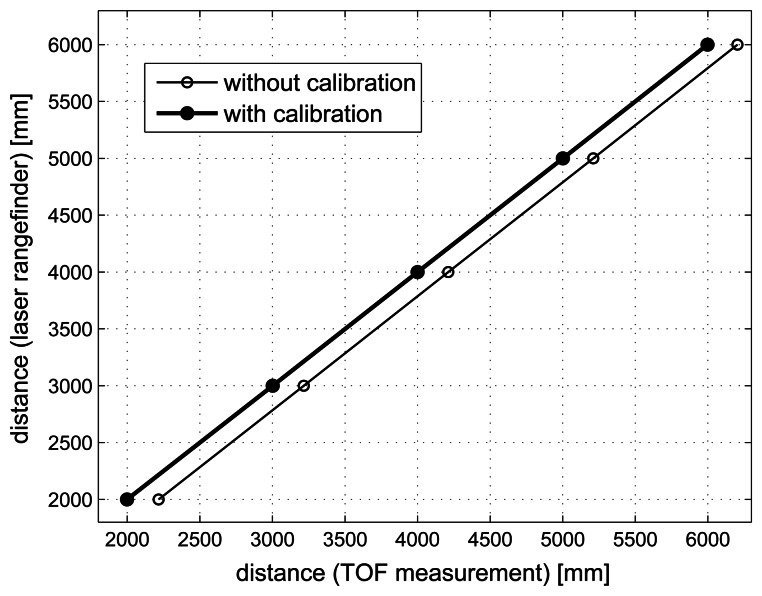
Calibration procedure for a pair of nodes. A linear regression of the distance measurements is used in order to compensate the group delay in the TOF measurements. The distances measured with the laser rangefinder are plotted on the Y-axis, while the averaged distances obtained from the TOF measurements are plotted on the X-axis. The thin line shows the linear regression of the plotted dots before the group delay compensation. Here a distance error of 22.41 cm is observed. The thick line shows the regression line obtained after calibration of the nodes.

Factors such as the signal filtering in reception or the calculation of the signal envelope at the output of the matched filter are the cause of a group delay that affects measurements (see Figure 6). The tolerance of the components used in the hardware design of the nodes leads to a different delay in each node. Therefore a calibration process of the nodes must be performed in order to compensate the group delay and to ensure a correct estimation of the TOF measurement. In this regard, we use a linear regression of the distance measurements obtained with our system using as reference the distances provided by the laser rangefinder. The group delay calibration is conducted for each pair of nodes. Figure 9 shows the calibration process for a particular pair of nodes. The distances measured with the laser rangefinder are plotted on the Y-axis. On the other hand, the averaged distances obtained from the TOF measurements (*d_TOF_*) are plotted on the X-axis. These values have been obtained using Equations (18) and (19). The linear regression of the plotted dots shows a line with a slope of *m* = 1.0029 and Y-intercept of *b* = −22.41 cm (thin line). These parameters are used to correct the distances inferred from the TOF measurements as

(23) $$\hat{d}=d_{\text{TOF}}\cdot m+b$$

The thick line shows the regression line obtained after calibration.

However, other factors can cause additional TOF estimation errors in a real environment; one of the most important is the multipath effect. The reflection of the ultrasonic signal in surrounding obstacles can create alternative propagation paths between emitter and receiver. In some situations, the multipath effect can cause the overlapping of two or more replicas of the ultrasonic signals at the receiver, modifying therefore the shape of the received signal. This shape modification will introduce an unknown bias in the TOF estimation obtained by the receiver matched filter. Dealing with such effects was not addressed in this work and we have used an experimental setup specifically designed to avoid them. When measuring the TOF between two network nodes, they were placed one in front of the other at a height of 1.6 m above the floor and 1.6 m farther away from walls and any other obstacle. For distances between emitter and receiver of less than 6 m, this setup ensures that any possible echo of the ultrasonic signal will reach the receiver at least 2.35 ms later than the main signal (the one propagated through the direct path). Considering that 1 ms ultrasonic signal was used in the experiments, this setup will avoid signal overlapping at the receiver due to multipath effects.

Table 2 shows the absolute error of the measurements (|*E*|) obtained with our system after calibration. The 90%, 95% and 99% percentiles of the absolute error are shown. The RMSE value and the maximum absolute error (|*E*|*_max_*) are also indicated. These errors were obtained by considering all measurements within the distance range of 2 to 6 m, assuming the following approaches on the TOF measurement of the ultrasound signal:

- TOF measurement obtained from the sampling instant in which the envelope reaches its maximum amplitude.
- Using parabolic interpolation for detecting the maximum of the signal envelope.
- Using parabolic interpolation and phase correction for detecting the maximum of the signal envelope at the matched filter output.

Different sampling frequencies were also used to prove the benefits of implementing of quadrature sampling, in particular for frequencies of 32.00 kHz, 17.78 kHz and 12.31 kHz (using *M* = 3, 5, 7, respectively). The results in Table 2 show a significant improvement in the accuracy of the system when a parabolic interpolation is applied to detect of the maximum of signal envelope. Similarly, although to a lesser extent, the phase correction contributes to improve the accuracy of the system. Another interesting conclusion of the results is the excellent accuracy of the system for sampling frequencies of 17.78 kHz or even 12.31 kHz. This justifies the use of quadrature sampling for optimizing the memory requirements and reducing the computational cost using a lower number of signal samples. For example, the total absolute error for Q = 99 is less than 3 mm using the B approach and a sampling frequency of 12.31 kHz. This error achieves a value less than 2.6 mm using the C approach. The average RMSE using this sampling frequency is lower than 2 mm for both approaches, with an absolute maximum error of less than 5 mm.

A portion of this absolute total error (|*E*|) is attributed to possible errors in the node positioning as well as the accuracy of the laser rangefinder whose measurements were taken as reference. A more interesting result can be the standard deviation value of the measurements. This parameter provides information on the repeatability of the measurements, regardless of the absolute error value. Table 3 shows the standard deviation (*σ*) of the measurements made at different distances using the A, B and C approaches for the sampling frequencies of 32.00 kHz, 17.78 kHz and 12.31 kHz.

For a distance of 6 m and a sampling frequency of 12.31 kHz, the results in Table 3 show a standard deviation less than 0.7 mm and 0.3 mm using the approaches B and C, respectively. This means that the system can achieve an accuracy of less than one millimeter, as long as an accurate calibration of the nodes is possible.

The clock time resolution of the TELIAMADE nodes is *T*_0_ = 1 *μ*s. When a transmitter node is synchronized with the coordinator node, the error between their respective clocks can be assumed uniform within the range ±T0/2 and the clock standard deviation will be $\sigma =T_{0}/\sqrt{12}$ (the same happens with the receiver node). The synchronization error between the transmitter and receiver is the difference of two independent uniform distributed random variables and will have a standard deviation $\sigma =T_{0}/\sqrt{6}$. This is equivalent to a distance error of 0.14 mm for a speed of sound of *c* = 340 m/s. Therefore the maximum accuracy of the system under ideal conditions is limited by this value. In Table 3, the results obtained from the B and C approaches are close to this minimum value, especially those obtained from the C approach. This demonstrates the goodness of the proposed quadrature detector and the success of the parabolic interpolation for detecting the envelope peak and also the phase correction to improve the accuracy on the TOF estimation. These results correspond to a scenario where the nodes are fixed. However, in real applications it is common for the receiver node to be moving.

**Figure 10.**
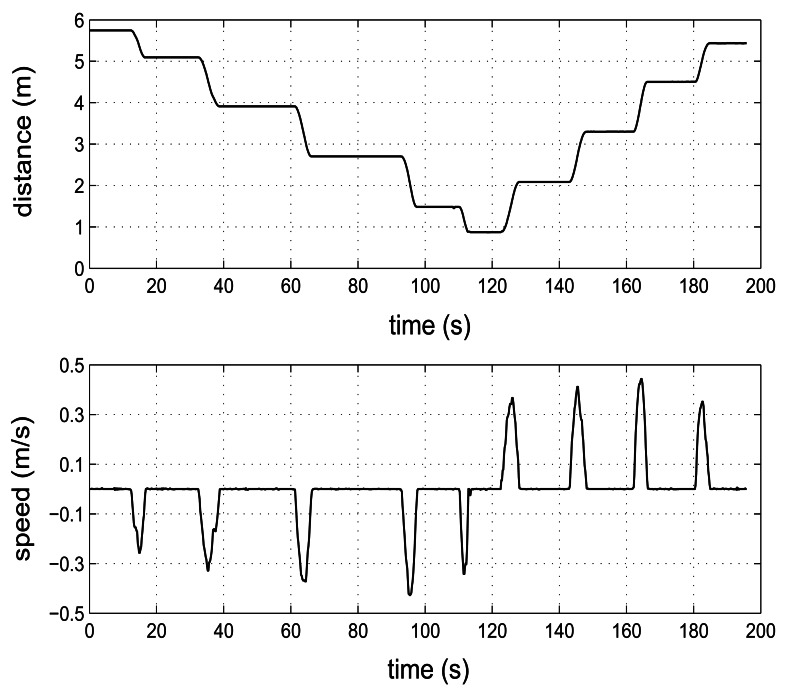
(Above) Dynamic estimation of the distance between a fixed transmitter node and a mobile receiver node. (Below) Dynamic estimation of the speed of the mobile node in its displacement from the transmitter node.

A second experiment was performed allowing the mobility of the receiver node while the transmitter node was fixed, in order to demonstrate the goodness of our system for estimating the distance in a dynamic way. In Figure 10 the estimated values of distance and speed for a moving receiver node from a fixed transmitter node are shown. The displacement of the receiver node was stepwise within the distance range of 2 to 6 m. For this experiment, the transmitter node was configured to transmit an ultrasonic pulse of 1 ms (using a rate of 5 measurements per second). A sampling frequency of 17.78 kHz (M = 5) and the B approach were used for estimating the TOF measurement in the receiver node. The above curve shows the displacement of the mobile node located initially at a distance about 6 m from the transmitter node. From this position the mobile initiated a movement of approaching to transmitter node and later a movement of distancing. The observed slopes in the distance curve (when the node is moved from one position to another) evidence the correct operation of the system in its dynamic estimate of the distance. The pauses between displacements show the repeatability of the measurements for a fixed position. The below curve in Figure 10 shows the speed changes arising from movement of the mobile node. In this case, the speed of sound was estimated at 346.49 m/s for an average temperature of 25.2 °C.

### 5.2. Positioning Accuracy by Applying Multilateration

**Figure 11.**
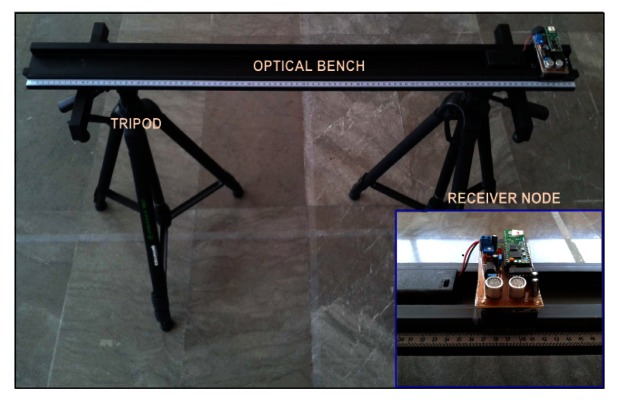
Test bench used to position the receiver node in different positions.

The positioning accuracy of the TELIAMADE system was analyzed in a clear room of dimensions (7 × 5) m^2^ and a height of 2.7 m. Four transmitter nodes were placed in the ceiling just in the center of the room with their ultrasound transducers pointing down. This deployment of the nodes avoids possible reflection of the signal on the nearby walls (multipath effect). The origin of the coordinate system was on the floor and coincides roughly with the center of the square formed by the four transmitter nodes when projecting their positions on the floor. The receiver node was located on an optical bench with its ultrasound transducers pointing up towards the ceiling. The optical bench was resting on two tripods (see Figure 11) to adjust the height of receiver node. The bench was located parallel to the axes and it was centered at the coordinate origin.

**Figure 12.**
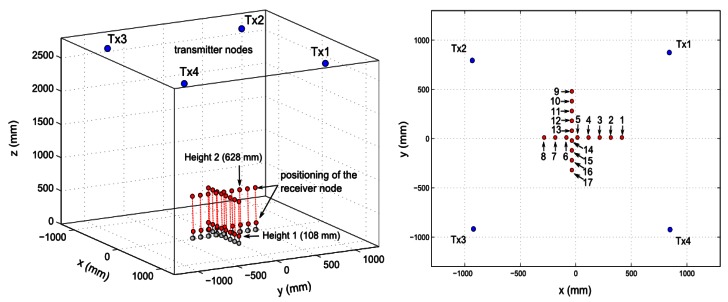
(Left) Illustration of the node position in the 3D-space. The red dots show the position of the receiver node on the optical bench. Positioning measurements are taken at two different heights (10.8 cm and 62.8 cm). (Right) Illustration of the node position in the 2D-space. The blue dots are the position of the transmitter nodes projected onto the xy-plane. The red dots are the positions of the receiver node in its displacement on the optical bench.

Positioning measurements in the direction of the x-axis and the y-axis were taken. To do so, the optical bench was placed parallel to the x-axis and then parallel to the y-axis. The receiver node was moved in 10 cm intervals on the bench. A total of 8 positions on the x-axis and 9 positions on the y-axis were evaluated (see Figure 12, right chart). Such measurements were conducted at two different heights, adjusting the tripods supporting the optical bench. These heights were 10.8 cm and 62.8 cm respectively (see Figure 12, left chart). A total of 300 positioning measurements were obtained at each position. The distances between the receiver node and the transmitter nodes were calculated by assuming an average temperature of 29.3 °C with a speed of sound of 348.87 m/s.

For this new experimentation, the transmitter nodes were configured to transmit an ultrasonic pulse of 1 ms. Such transmission was done in an orderly manner in order to avoid signal collisions in transmission using a TDMA approach. The network nodes were programmed to start the transmission or reception of ultrasonic pulses at given time instants. In this case, the channel is reserved during 200 ms to each transmitter node. Thus a cycle of four measurements is completed every 800 ms. The position of the receiver node was estimated by using multilateration from the distance measurements between it and the transmitter nodes. A sampling frequency of 17.78 kHz (M = 5) was fixed in the receiver by using the B approach for estimating the TOF measurement. The distance measurement was calculated considering the average value of the room temperature recorded in diverse positions, which was used for estimating of the speed of sound. Its value was 28 °C.

**Figure 13.**
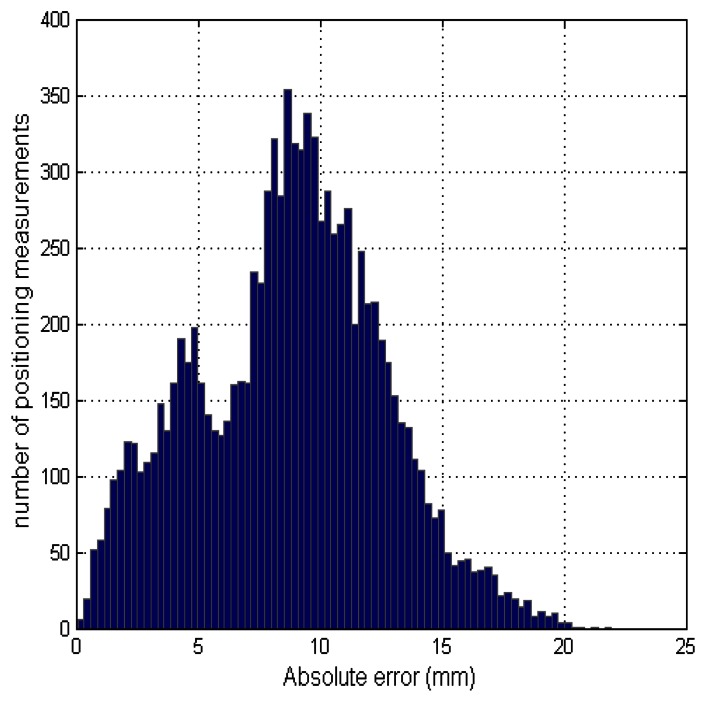
Absolute error histogram using all positioning measurements from both heights. The absolute error is calculated as the Euclidean distance between the estimated location and the true location.

Table 4 shows the average value of the positioning error in the x, y and z directions. These errors are obtained by subtracting the estimated coordinates of the receiver node with the reference coordinates for each position. The root mean square error (RMSE) of the positioning measurements taken at each position is also shown. These results indicate a sub-centimeter accuracy of the system in most of the assessed positions. The last row in the table shows the average absolute error from all measurements taken at the different positions for each height, where the average values of RMSE are less than 1 cm. Figure 13 shows the error histogram of the measurements taken in all positions for both heights. This error is calculated as the Euclidean distance between the estimated location and the true location.

Note that the histogram shape roughly corresponds to a mixture of two Gaussian distributions. Each distribution is the result from considering the positioning measurements taken at a certain height. The distribution of the measurements taken from height 2 has an average error greater than the distribution of measurements taken from height 1. This is because of the greater proximity between the receiver and the transmitters, resulting in a greater angle of emission or reception of the ultrasonic transducers. Since they are not omnidirectional transducers, the TOF measurement of the signal can be affected. Considering the contribution of both distributions, the histogram shape can be interpreted as a Gaussian distribution with a mean of 9.6 mm and a standard deviation of 3.8 mm.

Although the positioning results show a sub-centimeter location accuracy of the system, part of the error is due to an inaccurate estimate of the coordinates of the transmitter nodes. These results can be improved by using a calibration mechanism to precisely determine the position of the transmitter nodes. The error could also be reduced by including information about the angle of incidence of the transducers.

## 6. Conclusions

In this work we present the characteristics of the TELIAMADE system, a versatile LPS based on a wireless ultrasonic sensor network. Our approach is oriented to provide a new architecture for low power consumption, sub-centimeter location accuracy, and greater network configuration flexibility by using low memory requirements and a reduced computational cost.

Network nodes have an RF module by which they can communicate using the ZigBee protocol. A master node connected to a PC sends control messages to configure the operation of the network nodes. The ZigBee protocol enables managing the network efficiently by implementing several strategies to reduce the power consumption of the system and extend the battery life of the nodes. The nodes have also a hardware module for the transmission and reception of ultrasound signals, so that they can operate as ultrasonic transmitters or receivers.

The distance between nodes in TELIAMADE is calculated from the TOF measurement of ultrasound signal using the speed of sound, whose value is estimated considering the ambient temperature. The TOF of signal is estimated by using a quadrature detector. In order to optimize the memory requirements and to reduce the computational cost of signal processing, a band-pass quadrature sampling scheme is used. This enables using sampling frequencies as low as 17.78 kHz or even 12.31 kHz. The use of parabolic interpolation for detecting the envelope maximum value combined with the phase correction allows estimating the TOF with an accuracy of better than one-half the set sampling period (*T_s_*). The experimental results show an excellent accuracy of the system in the distance measurement using low sampling frequencies at a range of distances from 2 to 6 m. An RMSE value less than 2 mm and a standard deviation less than 0.3 mm are achieved from the distance estimates.

The mobile node position is calculated by applying multilateration from the estimated distance to the fixed nodes, which means that the location error will be affected by the distance errors. However, we think that other factors such as inaccuracies in the location of the fixed nodes or the angle of incidence of the ultrasonic transducers could possibly contribute to positioning error. Some experimental results show a location accuracy with RMSE values less than 1 cm. Therefore, we can say that the TELIAMADE system has a sub-centimeter location accuracy in an environment free of multipath effect on the ultrasound signal.

The system location area is limited by the angle of incidence and range of the ultrasonic transducers. In the current implementation, such area covers approximately 3 m^3^. In a future work, we would like to use omnidirectional transducers of greater range. This is feasible for the receiver as there exist MEMS-based sensors that are intrinsically omnidirectional [28].

## Figures and Tables

**Table 1.** Interpolated in-phase and quadrature components using an odd value for *M*.

***Comp.***	***m*** = **0**	***m*** = **1**	***m*** = **2**	***m*** = **3**
*Î*(*t_n_*)	+*x̂_i_*(*t_n_*)	+*x̂_q_*(*t_n_*)	−*x̂_i_*(*t_n_*)	−*x̂_q_*(*t_n_*)
*Q̂*(*t_n_*)	+*x̂_q_*(*t_n_*)	−*x̂_i_*(*t_n_*)	−*x̂_q_*(*t_n_*)	+*x̂_i_*(*t_n_*)

**Table 2.** The 90%, 95% and 99% percentiles of the total absolute error (|E|), RMSE value and maximum absolute error (|E|*_max_*) obtained for the sampling frequencies of 32.00 kHz, 17.71 kHz and 12.31 kHz. The values shown in the table correspond to the error obtained by considering all TOF measurements within the distance of range 2 to 6 m (2,r 500 measurements). Different approaches on the TOF measurement are used. Case (A): TOF measurement obtained from the sampling instant in which the envelope reaches its maximum amplitude. Case (B): Using parabolic interpolation for detecting the maximum of the signal envelope. Case (C): Using parabolic interpolation and phase correction for detecting the maximum of the envelope.

***Samp. Freq.***	***Case***	|***E***| (***mm***)			***RMSE*** (***mm***)	|***E***|*_max_* (***mm***)

		(***Q*** = **90**)	(***Q*** = **95**)	(***Q*** = **99**)		
32.00	A	7.678	7.754	8.245	4.500	8.921
(kHz)	B	3.300	3.469	3.710	2.110	4.172
	C	2.247	2.347	2.567	1.692	7.528

17.78	A	11.824	11.841	12.704	7.170	13.956
(kHz)	B	5.338	5.456	5.642	3.424	5.854
	C	2.245	2.388	2.676	1.613	4.676

12.31	A	15.528	15.629	15.909	9.982	16.332
(kHz)	B	2.533	2.702	2.961	1.497	3.224
	C	2.133	2.294	2.564	1.387	4.954

**Table 3.** Standard deviation (*σ*) of the measurements made at different distances using the A, B and C approaches for the sampling frequencies of 32.00 kHz, 17.78 kHz and 12.31 kHz. At least 500 measurements for each distance, sampling frequency and approach are taken.

***Samp. Freq.***	***Case***	***σ***(***mm***)				

		***d*** = **2 *m***	***d*** = **3 *m***	***d*** = **4 *m***	***d*** = **5 *m***	***d*** = **6 *m***
32.00	A	0.184	0.255	0.373	0.556	0.507
(*kHz*)	B	0.196	0.312	0.344	0.320	0.493
	C	0.246	0.301	0.271	0.436	0.278

17.78	A	0.484	0.427	0.346	0.541	0.580
(*kHz*)	B	0.217	0.348	0.318	0.425	0.520
	C	0.282	0.285	0.268	0.388	0.276

12.31	A	1.067	0.403	0.738	0.533	1.022
(*kHz*)	B	0.234	0.300	0.400	0.464	0.623
	C	0.258	0.318	0.369	0.435	0.282

**Table 4.** The average location error of the measurements taken at each position. The ex, ey and ez values are respectively the errors in the x-axis, y-axis and z-axis obtained by subtracting the estimated coordinates of the node with the true coordinates for each position. The global location error is calculated from these errors as the root mean square error (RMSE) for the measurements taken at each position. The last row in the table shows the average absolute error from all measurements taken at the different positions for each height.

	*Height*1 (108 *mm*)				*Height*2 (628*mm*)			

*Pos*	*ex* (*mm*)	*ey* (*mm*)	*ez* (*mm*)	***RMSE*** (*mm*)	*ex* (*mm*)	*ey* (*mm*)	*ez* (*mm*)	***RMSE*** (*mm*)
1	-1.2	-1.3	0.5	2.9	−5.3	9.8	5.0	12.4
2	0.5	−4.1	−0.8	4.9	14.7	−1.3	1.1	14.9
3	9.4	−5.2	0.0	10.9	−7.7	−2.5	−4.0	9.2
4	5.0	−4.2	0.6	7.0	−11.1	2.8	−3.7	12.2
5	−1.2	0.8	−0.5	2.4	0.1	10.5	1.3	10.8
6	3.4	−11.4	−2.1	12.2	2.3	3.4	0.4	4.4
7	3.1	5.3	−0.4	6.4	6.1	−6.2	2.9	9.4
8	−7.0	9.7	−3.6	12.7	−1.6	3.0	0.7	4.1
9	−8.3	−3.4	1.0	9.4	−4.0	−7.3	−0.4	8.6
10	0.2	−1.7	−0.3	2.6	−6.1	−3.7	−3.6	8.3
11	−9.7	0.1	1.8	10.1	9.3	−1.1	−1.0	9.7
12	4.4	6.1	3.1	8.5	7.1	2.4	0.5	7.8
13	8.8	−2.1	4.7	10.5	7.3	5.2	−0.3	9.1
14	8.5	−6.8	5.3	12.3	5.6	9.9	−1.4	11.6
15	−2.1	−3.9	2.2	5.4	1.1	0.2	−4.0	4.6
16	−15.9	−6.1	−0.8	17.2	−7.6	10.8	−0.2	13.3
17	−0.0	−7.9	−2.6	8.7	−7.7	1.2	−2.4	8.4

ALL	5.4	4.8	1.8	9.3	6.3	4.9	2.0	9.8

## References

[1] Sahinoglu Z., Gezici S., Güvenc I. (2008). Ultra-Wideband Positioning Systems: Theoretical Limits, Ranging Algorithms, and Protocols.

[2] Jekabsons G., Kairish V., Zuravlovs V. (2011). An analysis of WiFi based indoor positioning accuracy. Sci. J. Riga Tech. Univ. (RTU).

[3] Alhmiedat T.A., Yang S.H. (2008). A ZigBee-based mobile tracking system through wireless sensor networks. Int. J. Adv. Mechatron. Syst..

[4] Mattos P. (2004). Acquiring sensitivity to bring new signals indoors. GPS World.

[5] Kai C., Pissinou N., Makki K. Cellular Network Location Estimation via RSS-Based Data Clean Enhanced Scheme.

[6] Baertlein H., Carlson B., Eckels R., Lyle S., Wilson S. (2000). A high-performance, high-accuracy RTK GPS machine guidance system. GPS Solut..

[7] González E., Prados L., Rubio A.J., Segura J.C., de la Torre A., Moya J.M., Rodríguez P., Martín J.L. (2009). A Robust Indoor Ultrasound Location System: Design and Evaluation.

[8] Harter A., Hopper A., Steggles P., Ward A., Webster P. (2002). The anatomy of a contex-aware application. Wirel. Netw.-WINET.

[9] Nissanka B.P., Anit C., Hari B. The Cricket Location-Support System.

[10] Hazas M., Hopper A. (2006). Broadband ultrasonic location systems for improved indoor positioning. IEEE Trans. Mobile Comput..

[11] Prieto J.C., Jimenez A.R., Guevara J.I. Subcentimeter-Accuracy Location through Broadband Acoustic Transducers.

[12] Prieto J.C., Jimenez A.R., Guevara J., Ealo J.L., Seco F., Roa J.O., Ramos F. (2009). Performance evaluation of 3D-LOCUS advanced acoustic LPS. IEEE Trans. Instrum. Meas..

[13] (2003). Wireless Medium Access Control (MAC) and Physical Layer (PHY) specifications for Low Rate Wire-less Personal Area Networks (LR-WPANs). IEEE standard 802.15.4-2003.

[14] Microchip 28/40/44-Pin Enhanced Flash Microcontrollers with 10-Bit A/D and nanoWatt Technology. http://ww1.microchip.com/downloads/en/DeviceDoc/39626e.pdf.

[15] Texas Instruments CC2420 2.4 GHz IEEE 802.15.4 / ZigBee-ready RF Transceiver. http://www.ti.com/lit/ds/symlink/cc2420.pdf.

[16] Prowave 400ST120/SR120 data sheet. http://www.prowave.com.tw/pdf/T400S12.pdf.

[17] Philips Semiconductors 74HC04, 74HCT04 Hex inverter datasheet. http://www.nxp.com/documents/datasheet/74HCHCT04.pdf.

[18] N. Semiconductor LMC6482IN. http://www.ti.com/lit/ds/snos674c/snos674c.pdf.

[19] Waters W.M., Jarrett B.R. (1982). Bandpass Signal Sampling and Coherent Detection. IEEE Trans. Aerosp. Electron. Syst..

[20] Brown J.L. (1979). On quadrature sampling of bandpass signals. IEEE Trans. Aerosp. Electron. Syst..

[21] Medina C., Segura J.C., de la Torre A. (2012). A synchronous TDMA ultrasonic TOF measurement system for low power wireless sensor networks. IEEE Trans. Instrum. Meas..

[22] Vaughan R.G., Scott N.L., White D.R. (1991). The theory of bandpass sampling. IEEE Trans. Signal Process..

[23] Helms H.D., Thomas J.B. (1962). Truncation error sampling theorem expansions. Proc. IRE.

[24] Gueuning F.E., Varlan M., Eugne C.E., Dupuis P. (1997). Accurate distance measurement by an autonomous ultrasonic system combining time-of-flight and phase-shift methods. IEEE Trans. Instrum. Meas..

[25] Queiros R., Alegria F.C., Girao P.S., Serra A.C. (2010). Cross-correlation and sine-fitting techniques for high-resolution ultrasonic ranging. IEEE Trans. Instrum. Meas..

[26] Blackstock D.T. (2000). Fundamentals of Physical Acoustics.

[27] Medina C., Segura J.C., de la Torre A. (2012). Accurate time synchronization of ultrasonic TOF measurements in IEEE 802.15.4 based wireless sensor networks. Ad Hoc Netw..

[28] Logan A., Yeow J.T.W. (2009). Fabricating capacitive micromachined ultrasonic transducers with a novel silicon-nitride-Based wafer bonding process. IEEE Trans. Ultrason. Ferroelectr. Freq. Control.

